# *STAT5B::RARα*-positive acute promyelocytic leukemia: Role of next generation sequencing in detection of a rare malignancy

**DOI:** 10.1016/j.htct.2024.07.009

**Published:** 2024-11-14

**Authors:** Indranil Dey, Sushant Vinarkar, Mayur Parihar, Deepak Kumar Mishra

**Affiliations:** a Department of Molecular Pathology, Molecular Genetics, Tata Medical Center, Kolkata, India; b Department of Laboratory Haematology, Tata Medical Center, Kolkata, India; c Department of Cytogenetics, Tata Medical Center, Kolkata, India

## Introduction

Acute promyelocytic leukemia (APL) accounts for approximately 8–12 % of all acute myeloid leukemia (AML) cases.^1^ The majority of APL patients harbor the canonical translocation t(15;17)(q22;q12–23) leading to promyelocytic leukemia::retinoic acid receptor alpha (*PML::RARα*) fusion transcripts. In fact, this hallmark translocation, present in >95 % of cases of APL, can be detected using cytogenetic and molecular diagnostic modalities such as karyotyping, fluorescence *in situ* hybridization (FISH), reverse transcription-polymerase chain reaction (RT-PCR) and next generation sequencing (NGS).^2^ With the advent of all-trans retinoic acid (ATRA) and arsenic trioxide (Arsenic Trioxide) in 1988 and 1996, respectively, as cornerstone drugs in the management of classical APL, the cure rates have improved immensely and mortality rates have drastically decreased.^3^^,^^4^

However, on rare occasions, morphologically and immunophenotypically diagnosed APL cases are associated with translocations other than the classic t(15;17). Such atypical APL cases may harbor translocations resulting in fusions between *RARα* and genes such as *PLZF, NuMA, NPM*, and *STAT5B*.^5^, ^6^, ^7^ Therapeutically, APL can be divided in two disease subtypes: the ATRA-responsive subtype, which includes *RARα* fusions with *PML, NPM1, NuMA*, and others; and a ATRA-unresponsiveness subtype characterized by the presence of the *ZBTB16::RARα* and *STAT5B::RARα* fusions.^8^ Patients with the ATRA-unresponsive variant exhibit dismal prognoses characterized by frequent relapses and recurrences.^9^^,^^10^ Here we report a new case of *STAT5B::RARα*-positive AML focusing on its diagnostic features and the important role of NGS in its definitive diagnosis.

## Case report

A 42-year-old hypertensive and diabetic male presented to the hospital with complaints of severe weakness, decreased appetite and loss of weight for three months. He also developed difficulty of vision in both eyes along with slurring of speech and right upper limb weakness for ten days. Upon examination, the patient was obese and hypertensive (blood pressure: 160/94 mmHg). He had moderate to marked pallor and mild splenomegaly.

**Figure 1:**
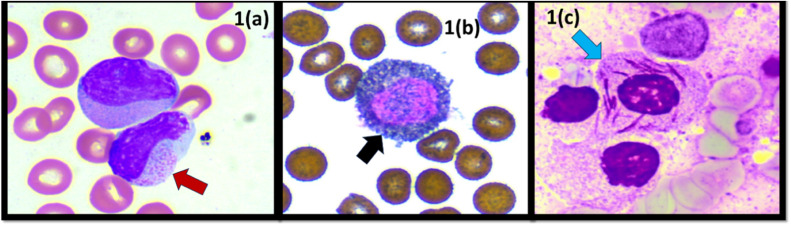
(a) Peripheral blood smear (PBS): blast/abnormal promyelocytes with salmon-pink cytoplasmic granules (red arrow). (b) PBS: blasts with strong cytochemical myeloperoxidase staining (black arrow - 40x). (c) Bone marrow aspirate: blasts with prominent Auer rods and faggot cells. (blue arrow - 40x).

His complete blood count upon evaluation showed a white blood cell count of 42.2 × 10^3^/µL along with thrombocytopenia (63×10^3^/μL) and anemia (8.9 g/dL). A peripheral blood smear (PBS) revealed 76 % blast/abnormal promyelocyte/atypical monocytoid cells with many showing salmon-pink cytoplasmic granules and strong cytochemical myeloperoxidase staining (Figure 1). In addition, the patient's coagulation parameters were deranged with persistently prolonged partial thromboplastin time and hypofibrinogenemia.

Flow cytometric immunophenotyping performed on peripheral blood revealed 60 % blasts/ abnormal promyelocytes with high side scatter, dim CD45, showing expression of CD117, CD13, CD33, CD56, and cMPO and negativity for CD34, and HLA DR. The PBS morphology and immune-phenotyping were suggestive of a diagnosis of APL.

**Figure 2:**
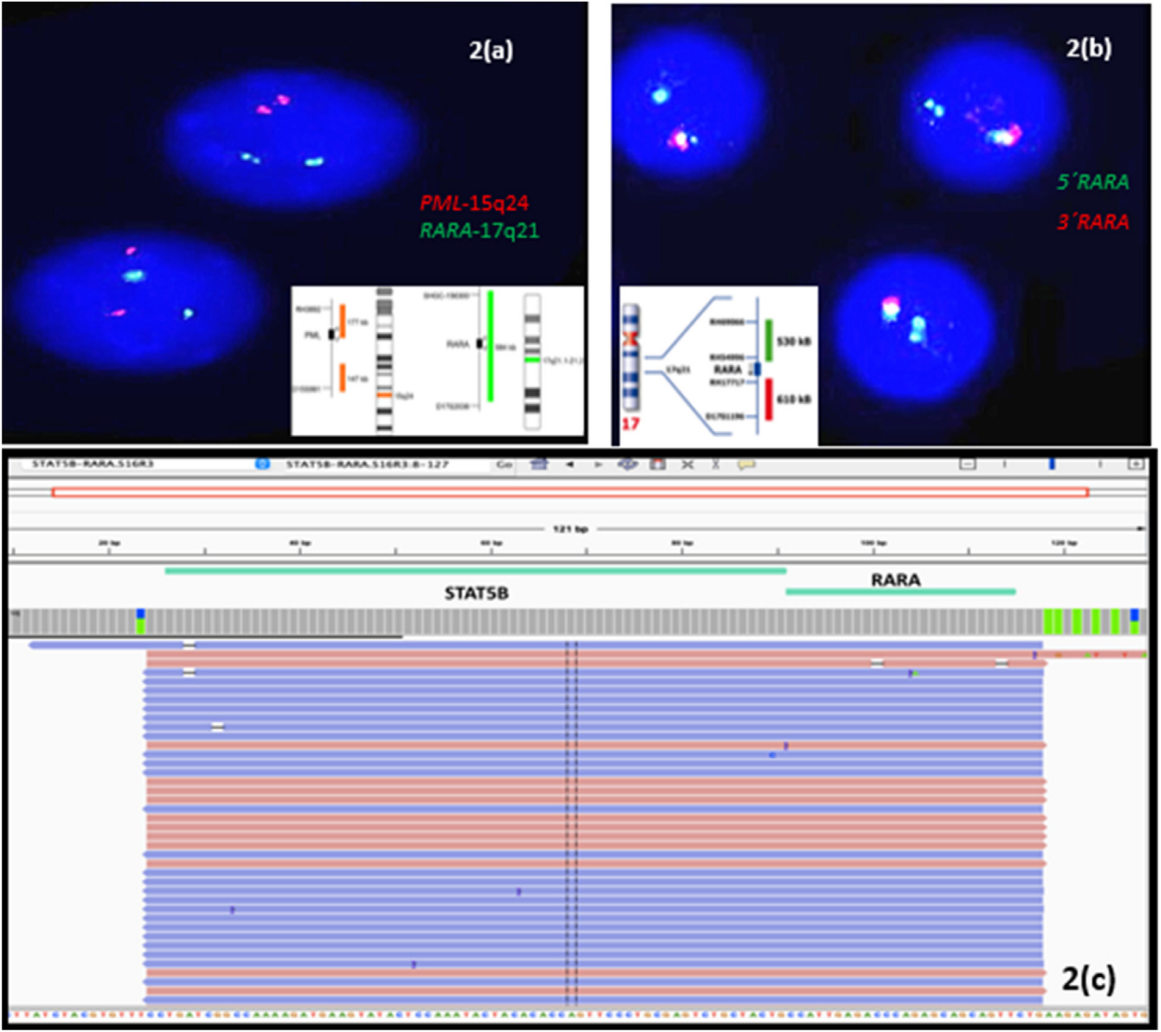
Cytogenetic and molecular genetic test findings. (a) Fluorescence *in situ* hybridization (FISH): *PML::RARα* dual color dual fusion probe: 2G2R - one weak green signal. (b) FISH:*RARα* break-apart probe: 1F1 G signal indicating deletion of areas distal to 3´ of *RARα* gene (interphase nuclei). (c) Integrative Genomics Viewer (IGV v2.11.2) showing *STAT5B::RARα* gene fusion on Ion Torrent Oncomine Myeloid Research Assay® (NGS) performed using the Ion GeneStudio™ S5 system.

Following these findings, cytogenetic studies were performed with FISH for the *PML-RARα* fusion gene using a dual-color, dual-fusion probe (MetaSystem, Germany). The FISH result was negative. However, a weak breakpoint signal (one light green signal) was detected and therefore, FISH was again performed utilizing the *RARα* break-apart probe (Kreatek, Leica, Amsterdam) which revealed an atypical *RARα* rearrangement (1F1 G signal pattern-Figure 2). These findings hinted towards the possible presence of a variant *RARA* translocation. Qualitative RT-PCR for *PML::RARα*, BCR1, BCR2 and BCR3 transcripts was also negative.

A bone marrow aspirate revealed a cellular marrow with 30 % myeloperoxidase positive blasts/ abnormal promyelocytes, many of which showed prominent Auer rods and occasional faggot cells (Figure 1). Overall, the bone marrow examination findings along with morphologic and immunophenotypic findings (absence of CD34 and HLA-DR) were consistent with APL.

As cytogenetic testing and RT-PCR was unable to pick up the variant RARA translocation, RNA and DNA sequencing was performed using the NGS-based comprehensive myeloid gene panel (Ion Torrent Oncomine Myeloid Research Assay®, ThermoFisher) assay. The Oncomine Myeloid Research Assay® is a targeted NGS panel which specifically detects relevant DNA mutations and RNA fusion transcripts associated with myeloid disorders and comprises 40 key DNA target genes and 29 driver genes in a broad fusion panel to cover all the major myeloid neoplasms. RNA sequencing (NGS) revealed a *STAT5B::RARα* fusion transcript (6094 read-counts – Figure 2). Additionally, the DNA sequencing (NGS) showed the presence of a missense gain-of-function variant in exon 5 of the *GATA2* gene (R362 G) [NM_032638.5:c1084C>G;p.(Arg362Gly)] with a variant allele frequency of 43.75 %.

The patient was started on ATO along with prophylactic steroid and hydroxyurea. Seven days after admission, the patient suddenly developed lower gastrointestinal bleeding and respiratory distress for which an emergency tracheostomy was performed. His condition further deteriorated and he suffered multiple cardiac arrests and ultimately expired after repeated attempts at resuscitation.

## Discussion

The signal transducer and activator of transcription 5B (*STAT5B*) gene is located on chromosome 17q21.2 and belongs to the Janus kinase (JAK)/STAT signaling pathway. It participates in intracellular signaling pathways, encodes transcription factors and regulates the proliferation and differentiation in hematopoiesis.^11^ *STAT5B::RARα* predominantly results from an inversion of 17p11.2 and 17q21.2 interstitial micro-deletion. Three different types of *STAT5B::RARα* transcripts have been reported, with breakpoints occurring in exon 14, exon 15, or exon 16 of *STAT5B* and in exon 3 of the *RARα* gene.^12^ The resulting fusion leads to deregulation of the JAK/STAT5 signal transducing pathways in leukemic cells and might explain the unusual features of the *STAT5B-RARα* APL variant.^13^

*STAT5B* also prevents further differentiation of hematopoietic cells together with the corepressor complex of deacetylase. This corepressor complex cannot be released by ATRA and this may explain the resistance of *STAT5B::RARα* APL to ATRA. Few cases have been reported to show an initial response to ATRA and ATO, however relapse and extramedullary infiltration is common in most cases. Conventional combination chemotherapy (daunorubicin and cytarabine (Cytarabine)/idarubicin) also does not fare much better in these patients, so haemopoietic stem cell transplantation (HSCT) appears to be the only effective treatment according to documented reports.^10^ The optimal timing for HSCT has been reported to be after achieving first complete remission (CR).

Resistance to the otherwise highly effective ATRA/ATO therapy makes it crucial to detect variant RARA gene partners in APL, especially since they are morphologically and immunophenotypically difficult to distinguish from the classic t(15;17)(q24.1;21.2) APL (*PML::RARα* gene fusion).^9^ In previously reported cases, most patients received ATRA alone (50 %) or in combination with ATO/chemotherapy (40 %) as first line treatment, however CR was achieved by only a fraction of these patients (10 % monotherapy and 40 % combination therapy) after the first course. The CR rates are markedly reduced in *STAT5B::RARα* APL as compared to classical *PML::RARα* APL patients (35.3 % and 95 %, respectively). These cases also have higher relapse and mortality rates as compared to *PML::RARα* APL (mortality rates: 47.1 % and 5 %, respectively). Infections, progressive disease, cerebral hemorrhage and transplant related complications were the most common causes of mortality.

Until now, only 17 cases of APL with *STAT5B::RARα* have been reported worldwide, making this a very rare entity (Table 1). To the best of our knowledge, this is the first reported case from the Indian subcontinent. Most of the previously reported *STAT5B::RARα* patients were middle aged (age range: 17–67 years with a mean age of 39 years). The majority of the patients were men, with women accounting for only two cases (11.76 %). More data is required to determine the significance of the age and gender distribution of these patients.

**Table 1:** Clinicopathological profile of STAT5B::*RARα* patients reported in literature.

#	Year of publication	Author's name	Age	Gender	WBC (x 10^9^)	PLT (x 10^9^)	Effective treatment	OS (months)
**1**	1996	Arnould C^10^	67	M	6.7	NA	None	NA
2	2008	Kusakabe M^5^	42	M	3.6	125	DA + Mit + VP16	21
3	2009	Iwanaga E^11^	41	M	77.8	95	ATRA + IA	17
4	2011	Jovanovic JV^12^	29	F	5.6	NA	ATRA + IA allo-BMT	15
5	2011	Qiao C^13^	32	M	3.8	282	FLAG allo-HSCT	–
6	2012	Chen H^14^	26	M	6.6	94	None	NA
7	2013	S. Strehl^15^	57	M	23.8	NA	DA+ gemtuzumab ozogamicin	18
8	2013	Strehl S^15^^,^^16^	17	M	2.8	NA	ATRA, IA, VP16	53
9	2015	Kluk MJ^17^	47	M	2.1	135	HSCT	9
10	2015	Wang YY^18^	22	M	2.3	125	None	9
11	2016	Lirong L^19^	49	M	19.7	24	ATRA + ATO + idarubicin IA - HHT + MD-Ara-C	>15
12	2017	Pessina C^20^	32	M	6.8	126	Allo-HSCT	12
13	2018	Zhang C^21^	28	F	1.95	196	None	2
14	2018	Wang A^22^	47	M	17.18	78	decitabine (Dacogen) + AA/IA	12
15	2019	Ciangola G^23^	47	M	1.6	229	FLAG-IA + ATRA	NA
16	2019	Peterson JF^24^	27	M	52	24	None	4 days
17	2020	Wang L^25^	62	M	1.83	54	ATRA ATO, idarubicin, decitabine (Dacogen) + CAG	8
18	2023	Present case	42	M	42.2	63	ATO + Hydrea + Steroids	7 days

OS, Overall survival; DA, Daunorubicin; Mit, Mitoxantrone; ATRA, all-trans retinoic acid; ATO, arsenic trioxide (Arsenic Trioxide); IA, Idarubicine; allo-BMT, Allogeneic bone marrow transplant; FLAG, Fludarabine, cytarabine (Cytarabine) and G-CSF; allo-HSCT, Allogeneic stem cell transplant; HHT, homoharringtonine; Ara-C, Cytosine arabinoside; CAG, cytarabine (Cytarabine), aclarubicin and G-CSF.

Due to the clinical urgency, it is justified to perform a comprehensive cytogenetic and molecular profiling of atypical APL patients utilizing *PML::RARα* FISH, RARa BAP FISH and molecular tests such as RT-PCR and NGS-based assays to detect these cases promptly. NGS-based assays can be utilized to characterize most structural abnormalities throughout the genome with significantly improved accuracy and precision as compared to conventional cytogenetics and FISH tests.^26^ NGS can effectively detect different RARA fusion partners such as *PML, STAT5B, ADAMTS17, NUMA1, FIP* *1L1, ZBTB16, PRKAR1A, BCOR, NPM1, TBL1XR1* and *NABP1*.

In addition to the *STAT5B-RARα* fusion, a missense mutation in the *GATA2* gene (R362 G) was detected in this patient. *GATA2* mutations usually present as germline variants and have been known to predispose to AML and myelodysplastic syndromes.^27^ *GATA2* mutations have also been reported in *de novo* AML, especially in adult patients with biallelic *CEBPA* mutations.^28^

By advanced functional and expression studies, it has been documented that the GATA2 gene is excessively expressed in *PML::RARα*-positive pre-leukemic cells. Furthermore, GATA2 gene variants are seen to be persistent in transformed APL cells. *GATA2* gene somatic mutations have also been detected in APL patients during disease progression. The increased regulation of *GATA2* may help to check the proliferation of *PML::RARα* positive leukemic cells, and consequently, inactivation of the *GATA2* gene by mutation (and/or epigenetic silencing) may accelerate disease progression in APL and in other forms of AML.^29^ Due to the demise of our patient, the germline mutation status of the *GATA2* variant could not be ascertained.

Measurable residual disease (MRD) monitoring has greatly improved therapeutic decision making in cases of *PML-RARα*-positive APL, however, data regarding MRD monitoring in patients with the *STAT5B-RARα* rearrangement is very limited. In developing countries and in centers with limited resources, MRD monitoring of such rare entities becomes even more challenging. However, flow cytometry, FISH, RT-PCR and NGS-based techniques should be explored for MRD monitoring in these patients as this can help to better assess treatment response.

In conclusion, this case describes the significant clinical, diagnostic and therapeutic differences between *STAT5B-RARα* t(17;17) and PML-*RARα* t(15;17) fusion-positive APL and highlights the role of molecular diagnostics, cytogenetics and flow cytometry in the identification of this rare APL variant. It also demonstrates the valuable role of NGS-based assays in reaching a definite diagnosis of such a rare malignancy that warrants immediate treatment in order to avert mortality.

## Conflicts of interest

The authors declare no conflicts of interest.
